# Ascending single-dose study of the safety, pharmacokinetics, and pharmacodynamics of CSTI-500, a novel monoamine triple reuptake inhibitor, first-in-human

**DOI:** 10.1007/s00213-025-06861-4

**Published:** 2025-08-11

**Authors:** Lieuwe Appel, Robert Risinger, Anders Wall, Harald Murck, Shuang Liu, Gunnar Antoni, Roger Lane

**Affiliations:** 1 Molecular Imaging and Medical Physics, Department of Surgical Sciences, Uppsala University, Uppsala, Sweden; 2 PET Centre, Department of Medical Imaging, Uppsala University Hospital, 75185 Uppsala, Sweden; 3 BioXcel Therapeutics Inc., 555 Long Wharf Drive, New Haven, CT 06511 USA; 4 Philipps-University Marburg, Marburg, Germany; 5 Murck-Neuroscience LLC Westfield, Westfield, NJ USA; 6 ConSynance Therapeutics, Inc., 11 University Place, Rensselaer, NY 12144 USA; 7 Department of Medical Chemistry, Uppsala University, Uppsala, Sweden; 8 Ionis Pharmaceuticals, Inc., 2855 Gazelle Court, Carlsbad, CA 92010 USA

**Keywords:** Monoamine triple reuptake inhibitor, SERT, NET, DAT, Safety, Tolerability, Pharmacokinetics, Brain PET, Monoamine transporter occupancy, SAD

## Abstract

**Rationale:**

Monoamine triple reuptake inhibitors (TRIs) inhibit central dopamine, norepinephrine, and serotonin transporters, restoring functional monoamine neurotransmission.

**Objectives:**

This clinical trial evaluated the safety, tolerability, and pharmacokinetics in healthy volunteers after single-ascending-doses (SAD) of the novel monoamine TRI CSTI-500. In addition, we estimated the peak and duration of striatal serotonin transporter (SERT) and dopamine transporter (DAT) occupancies, by using positron emission tomography (PET).

**Methods:**

Part A was a double-blinded, randomized, placebo-controlled, sequential SAD study with seven sequential dose panels (0.5-150 mg) where subjects in each panel received either a single oral dose of CSTI-500 (*n=*6) or placebo (*n=*2). Part B was an open-label, single-dose PET study to assess the peak and duration of SERT (*n=*4) and DAT (*n=*5) striatal occupancies, using the radioligands [^11^C]MADAM and [^11^C]PE2I, respectively.

**Results:**

The maximum tolerable acute single-dose of CSTI-500 was determined as 100 mg. No serious adverse events occurred. The median maximum CSTI-500 concentrations were attained at 1-2 hours post-dose (h pd); the estimated plasma elimination half-life was 44-50 h pd. Subsequent to a single-dose of 100 mg CSTI-500, mean striatal SERT occupancy was 72% and 62% at 4-6 and 24 h pd, respectively. Mean striatal DAT occupancy was 36% and 31% at 4-9 and 24 h pd, respectively.

**Conclusions:**

CSTI-500 is a potent monoamine TRI with substantial striatal SERT and moderate DAT occupancies in healthy subjects. Together with promising safety-tolerability and pharmacokinetics profiles, the continued clinical development of CSTI-500 is strongly supported.

**Supplementary Information:**

The online version contains supplementary material available at 10.1007/s00213-025-06861-4.

## Introduction

Traditionally, a strong focus of drug discovery has been to develop drugs acting with high potency and selectivity against a specific biological target based on a cause-and-effect relationship paradigm (Anighoro et al. 2014). Conversely, drugs targeting a broad and sometimes unpredictable spectrum of biological activities might cause unacceptable adverse events (AEs). Today, however, these concepts are increasingly considered an inadequate approach to complex multifactorial and polygenic diseases. As a result, combination and augmentation therapies have become common in clinical settings, varying from a cornerstone in cancer treatment (Bayat Mokhtari et al. 2017) to a common need in the treatment of major depression based on individual assessment (Culpepper et al. 2015; Kato and Chang 2013; Moret 2005). The highly variable pharmacokinetics of psychotropic drugs between individuals is a particular challenge, as individually optimizing the pharmacokinetics and pharmacodynamics of different drugs is a complicated task. One drug acting with optimized relative affinities on several targets may result in a clearly understood profile of pharmacodynamic effects across and within individuals. Further, a one-pill concept is a comprehensible and rational approach to dose adjustment and, thus, to achieve appropriate pharmacodynamic effects, which may result in adequate clinical efficacy and fewer AEs. Improving the balance between benefits and harms will enhance the patients´ adherence to medication in daily clinical practice. However, developing multi-target drugs with appropriately tuned pharmacological properties has hitherto represented a significant challenge in drug discovery, particularly in medicinal chemistry when therapeutic targets belong to the same family and exhibit substantial homology (Tu et al. 2021).

Monoamine triple reuptake inhibitors (TRIs), targeting central transporters for serotonin (SERT), norepinephrine (NET), and dopamine (DAT), have been investigated for various central nervous system (CNS) disorders related to the dysfunction of these monoamine systems. Several monoamine TRIs have been aimed at the treatment of patients with major depression (MDD), as a large proportion of patients do not experience remission of symptoms but suffer from undesired, long-lasting side effects after treatment with conventional single (SERT) and dual (SERT/NET) monoamine targeted antidepressants (Cipriani et al. 2018; Fava and Rush 2006). In MDD, the inhibition of SERT and NET, enhancing levels of serotonin and norepinephrine, is aimed at mitigating negative mood states associated with distress, which are heightened in this disorder (Lane 2014). On the other hand, increased functional dopamine activity by an inhibitory effect on dopamine transporters may cause a rehabilitation of positive mood symptoms associated with well-being, which are decreased in MDD. Given that a variety of neuropsychiatric disorders are often comorbid with MDD (Millan 2009) and may share, at least in part, neural network perturbations, treatment with monoamine TRIs might be helpful in the management of several comorbid neuropsychiatric disorders comprising many different etiologies.

CSTI-500 (also known as BMS-866949) is a novel, competitive, and selective TRI of SERT, NET, and DAT, primarily developed for the symptomatic treatment of MDD by Bristol-Myers Squibb (BMS). Previously, this monoamine TRI was extensively evaluated in a series of *in vitro* and *in vivo* preclinical experiments (data on file). CSTI-500 was demonstrated to saturate recombinant human SERT, NET, and DAT completely, and at neurotransmitter binding sites, this potent binding resulted in average IC50 values of 1.9, 23, and 34 nM, respectively, as determined using [^125^I]-RTI-55 for SERT and DAT, and [^125^I]−2-iodo-nisoxetine for NET. *In vivo* CSTI-500 was effective in a dose-dependent manner in the mouse tail suspension model of depression with the lowest effective dose at 10 mg/kg orally, resulting in occupancy values of 82%, 54%, and 65% for SERT, NET, and DAT, respectively, as measured *in ex vivo* binding experiments. A time-course occupancy study in mice, after a single-dose of 5.6 mg/kg CSTI-500, showed extended exposure near the peak plasma concentration (1305 nM) from 1 to 8 hours (h) post-dose (pd) with peak occupancies for SERT, 84% at 1 h; NET, 51% at 1 h and DAT, 53% at 5 h. At 24 h pd, mean plasma levels of CSTI-500 had decreased to 117 nM, and the occupancies declined to approximately: SERT, 47%; NET, 25%; DAT, 17%. Various *in vivo* animal experiments showed that the distribution of CSTI-500 into the brain was high, consistent with good *in vitro* membrane permeability and limited P-glycoprotein (P-gp) mediated efflux. Sixteen metabolites of CSTI-500 were observed collectively during *in vitro* and *in vivo* studies based on diverse biotransformation routes. Detected brain metabolites exerted monoamine transporter inhibition of SERT, NET, and DAT but with far less potency in the brain than the parent compound (>500-fold higher EC_50_ values).

For the successful development of monoamine TRIs, a critical factor is achieving an optimal balance of inhibitory potencies across the transporters. As such, *in vivo,* occupancy time profiles in humans, assessed using positron emission tomography (PET), are invaluable for exploring TRIs potency to block monoamine transporters and their relationship to safety, tolerability, pharmacokinetics, and consequently offering a basis for decisions in drug development, such as indication, dose and its therapeutic potential (Appel et al. 2014; Comley et al. 2013; DeLorenzo et al. 2011; Matuskey et al. 2023; Risinger et al. 2014; Talbot et al. 2010; Zheng et al. 2015). To date, the most promising results for monoamine TRIs have come from studies of tesofensine for obesity treatment (Astrup et al. 2008; Huynh et al. 2022) and centanafadine for attention-deficit/hyperactivity disorder (ADHD) (Adler et al. 2022; Wigal et al. 2020). In 2013, BMS decided to cease drug development for CNS-related disorders for strategic reasons soon after completing the first clinical studies for CSTI-500. This decision included a termination of BMS’s extensive program for developing monoamine TRIs for psychiatric disorders, which included treatment-resistant depression (Cuss 2015). CSTI-500 continues to be developed by ConSynance Therapeutics, Inc. as a pharmaceutical treatment for rare obesity disorders associated with hypothalamic dysfunction, such as Prader-Willi Syndrome and hypothalamic-injury-induced obesity.

This first-in-human phase I study evaluated primarily the safety, tolerability, and pharmacokinetics (PK) after single-ascending-doses (SAD) of CSTI-500. Secondly, PET imaging with highly selective radioligands was conducted to investigate the peak and duration of SERT and DAT occupancy, particularly for the highest safe and tolerable dose. Relationships between transporter occupancies, PK, and potential drug-related AEs were explorative. A commonly accepted, valid and reliable method was unavailable for quantifying NE transporter occupancy by using PET (Logan et al. 2007; Severance et al. 2007) when the study was initiated (2009); therefore, the determination of NET occupancy was excluded. The outcome of this clinical trial was expected to give early insights into the properties of this monoamine TRI and its therapeutic potential in humans and consequently support decisions for subsequent studies. In addition, our results may invigorate the development of novel therapies for disorders with monoamine dysfunctions and allow comparisons with similar target agents. This study also promotes further interest in the neurochemistry of neurological disorders, including developing highly selective PET tracers with novel clinical applications.

## Methods

### Study design

The presented work was a two-part phase I study in healthy subjects with different main objectives and a distinct design for each part. The first part (A) was a SAD study that evaluated safety, tolerability, and for single oral doses ranging from 0.5 to 150 mg. The second part (B) was a single-dose PET study using the highly selective radioligands [^11^C]MADAM and [^11^C]PE2I to assess striatal SERT and DAT occupancy profiles. In part B, the selected single-dose of CSTI-500 was based on the outcome of part A.

The SAD study had a double-blinded, randomized, placebo-controlled, sequential design. Both sexes could be included, but only women without childbearing potential as no preclinical reproductive toxicology data was available. Eight subjects each were assigned to seven sequential dose panels (0.5, 2.5, 10, 25, 50, 100, 150 mg), receiving either a single oral dose of CSTI-500 (*n=*6) or a matching placebo (*n=*2). Subjects were fasted at least 10 h before and 4-5 h after dosing. Decisions for dose escalation were based on reported AEs, vital signs, ECGs, tolerability (up to 72 h pd), and clinical laboratory results (48 h pd) of the preceding dose panel for subjects administered CSTI-500.

The PET study had an open-label and sequential design to explore the time course of central SERT and DAT occupancy after a single-dose of CSTI-500. Only males were eligible to exclude possible confounding effects of sex (SERT Jovanovic et al 2008; Silberbauer et al. 2022); (DAT Munro et al. 2006; Varrone et al. 2013). Restricting participation to a single sex helps limit the inter-subject variability of the dose-occupancy relationship, which is a critical factor for enabling conclusions in cases with small cohorts. Subjects were assigned randomly to a SERT or DAT cohort for examinations using [^11^C]MADAM PET or [^11^C]PE2I PET, respectively. The radioligands [^11^C]MADAM and [^11^C]PE2I are validated and recognized as highly selective for SERT (Chalon et al. 2003; Halldin et al. 2005; Larsen et al. 2004; Lundberg et al. 2005) and DAT (Chalon et al. 1999; Emond et al. 1997; Guilloteau et al. 1998; Halldin et al. 2003), respectively. For both cohorts, a baseline PET scan prior to dosing and two pd PET scans were scheduled to assess SERT and DAT occupancies’ peak and duration. Subjects were required to fast at least 10 h before dosing and 4 h before each PET scan. However, they got a short, light breakfast 1 h after dosing, i.e., 3 h before the first pd PET scan. The PET study design initially comprised two dose panels with up to 12 subjects in each panel. The first panel should determine the SERT and DAT occupancy profiles of the maximum tolerable single-dose (MTD) identified in the SAD study. Further, there was an option for a second panel to examine occupancies for various CSTI-500 doses lower than MTD.

Screening of subjects was done within 30 days before study enrolment. Subjects were admitted to the clinical facility on Day-1 and remained there until Day 7. A final visit to the facility was made on Day 14 for discharge.

The study was performed with approvals from the Swedish Medical Products Agency, the local Independent Ethics Committee, and the local Radiation Protection Committee. All subjects signed informed consent before enrolling in the study and after receiving a comprehensive explanation of study procedures. The conduct of the study followed the ethical principles of the current Declaration of Helsinki, International Conference on Harmonisation for Good Clinical Practice, and applicable regulatory requirements. This clinical trial was registered as NCT00949767 at ClinicalTrials.gov.

### Participants

In the SAD study, 56 subjects were enrolled with an average age of 25 (range 18-55 years) and a BMI of 23.5 (range 19.0-29.4 kg/m^2^). Enrolment was based on medical history and results of physical examination, 12-lead electrocardiogram (ECG), and clinical laboratory tests in blood and urine. Subjects were free from any illness, disorder, medication, or addiction that could affect the study outcome, following detailed inclusion and exclusion criteria and judgments by the principal investigator. Further, in the absence of preclinical reproductive toxicology data, only women without child-bearing potential were eligible to participate.

In the PET study, nine subjects participated, and the average age and BMI were 25 (range 21-29 years) and 25.4 (range 24.3-28.1 kg/m^2^), respectively. Imaging related exclusion criteria were: subjects had previously participated in a PET study or undergone any clinical procedure involving significant exposure to ionizing radiation, and subjects with contraindications for brain magnetic resonance imaging (MRI) and PET procedures. MRI examinations (T1-IR, T2-weighted, and Flair) were conducted on a 1.5-Tesla scanner (Philips Healthcare, Best, The Netherlands) during the screening period, and subjects with clinically significant brain abnormalities were excluded.

**Fig. 1:**
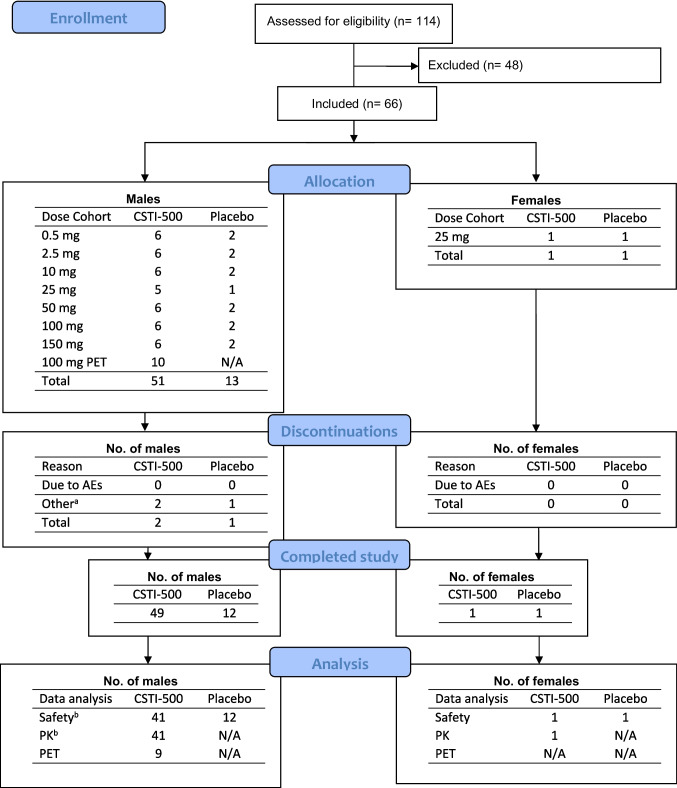
CONSORT flow diagram. Notes: ^a ^Discontinuations in the SAD study (part A) were due unfulfillment of the final follow up (day 14) and stoppage of dosing before treatment in the 100 mg and 150 mg dose panels, respectively. In the PET study (part B) one subject withdrew his consent to participate prior to dosing. ^b ^Inclusion of all treated subjects that completed the study until day 7.AE, adverse event; PET, positron emission tomography; PK, pharmacokinetics; SAD, single ascending dose

A CONSORT flow diagram (Fig. 1) summarizes the process from screening to study completion and the data available for analysis.

### Assessments

#### Safety and tolerability

Safety and tolerability assessments were acquired pre- and post-dose throughout the study and were primarily based on the medical review of AEs. In addition, vital sign measurements (heart rate and blood pressure), ECGs, physical examinations, and clinical laboratory tests (blood and urine) were evaluated. Data were compared to baseline assessments and, if applicable, to placebo.

In the SAD study, heart rate and blood pressure were recorded continually at 30-min intervals from 0.5–15 h pd by an automated ambulatory monitor ((ABPM; Spacelabs Healthcare; Snoqualmie, WA, USA) to provide time-course data for evaluating any potential cardiovascular effect of CSTI-500. After completion of the study, it was noticed that operational procedures related to fasting (from 10 h before dosing) and food consumption (4-5 and 10 h pd) affected the measurements, particularly heart rate, but also notable for blood pressure. For details, see Online Resource 1. Consequently, only data from stable fasting conditions (2-4 h pd) were utilized to evaluate an acute effect of CSTI-500 on the vital sign measurements.

#### Pharmacokinetics

The SAD study was designed to examine the PK profiles for various doses of CSTI-500. Therefore, serial venous blood samples were collected frequently up to 16 h after administration on Day 1, and one discrete blood sample on Day 2, 3, 4, and 7, resulting in a 144 h pd time interval. In the PET study, one venous blood sample was taken promptly before and after the pd PET scans to investigate, exploratively, the relationship between plasma CSTI-500 concentrations and occupancies estimated by PET. In addition, discrete venous blood samples were taken at 4, 7, 48, 72, and 144 h after study drug administration to verify the results with those from the SAD study.

CSTI-500 concentrations were determined in plasma following a validated liquid chromatography-tandem mass spectrometry (LC-MS/MS) procedure. The derived PK parameters were maximum observed plasma concentration (C_max_), time to C_max_ (T_max_), the concentration at 24 h after dosing (C_24h_), the area under the plasma concentration-plasma time curve from zero to the time of the last quantifiable concentration (AUC_0-T_) and extrapolated to infinite time (AUC_INF_), estimated plasma elimination half-life (T_1/2_) and oral plasma clearance (CLT/F).

#### Positron emission tomography

The radioligands were synthesized following reported methods for [^11^C]MADAM (Tarkiainen et al. 2001) and [^11^C]PE2I (Halldin et al. 2003; Hirvonen et al. 2008). For each subject, three PET scans were scheduled on three consecutive days following the dosing of CSTI-500: one baseline scan (day before dosing), 4 h pd (day 1), and 24 h pd (day 2). The PET investigations were performed on two identical and cross-calibrated ECAT EXACT HR+ PET scanners (Siemens/CTI, Knoxville, USA), which enabled the acquisition of 63 contiguous data planes with a distance of 2.46 mm. The subjects were positioned on the PET scanner couch with their head suitably fixed to prevent excessive movement. Before radioligand administration, a 10-min transmission scan was conducted in a two-dimensional mode for attenuation correction during image reconstruction. The radioligands were administered intravenously as a fast bolus via a venous cannula in the arm of the subject. The injected dose of radioactivity was on average 292 MBq (range 185-340; *n=*12) for [^11^C]MADAM and 321 MBq (range 280-337; *n=*13) for [^11^C]PE2I. The average injected mass was 2.1 µg (range 1.0-5.6) and 3.8 µg (range 1.8-7.6) for [^11^C]MADAM and [^11^C]PE2I, respectively. Simultaneously with the tracer bolus, an 80-min dynamic scan consisting of 22 frames with progressive frame duration (4x60s, 2x120s, 4x180s, 12x300s) was started. The PET data was reconstructed using Ordered Subset Expectation Maximization (OSEM), including 6 iterations and 8 subsets, using a 4 mm Hanning filter, resulting in a transaxial spatial resolution of about 5-6 mm in the field of view. All necessary corrections were applied according to the software supplied by the manufacturer.

The dynamic PET data were realigned within and between scans to adjust for movements during scanning and different positions in the scanner, respectively. The structural T1-IR MRI sequence was co-registered with the realigned PET scans to provide anatomical information for an appropriate delineation of volumes of interest (VOIs). The primary evaluated VOI was bilateral striatum, comprising nucleus caudate and putamen, regions with a significant SERT and DAT density. Further, the striatum is a distinct and substantial brain structure with a homogeneous tracer distribution, generally resulting in high reproducibility. Cerebellum was delineated as a reference region as it has no specific binding of [^11^C]MADAM (Halldin et al. 2005; Lundberg et al. 2005) and [^11^C]PE2I (Guilloteau et al. 1998; Hall et al. 1999; Halldin et al. 2003) due to negligible amounts of SERT and DAT, respectively. Pre-processing and VOI-delineation were conducted using VOIager software (GE Healthcare, Uppsala, Sweden).

Regional time-activity data were generated as input functions for the tracer kinetic analyses implementing a simplified reference model (Lammertsma and Hume 1996). Average time activity curves (TAC) are presented in Online Resource 2 for [^11^C]MADAM and [^11^C]PE2I. Kinetic modeling parameters were obtained by a non-linear least squares method using in-house developed MATLAB routines. The fit of the [^11^C]MADAM and [^11^C]PE2I TAC data is demonstrated for one subject, respectively, in Online Resource 3. For all subjects, the patterns were similar. Individual SERT and DAT occupancies were determined as the relative difference between the non-displaceable binding potential (BP_ND_) in the baseline and the pd scans. Two experienced raters blinded to the treatment allocation conducted the PET data analysis.

## Results

### Single-dose escalation study

#### Dataset

In the SAD study, 55 subjects received treatment, CSTI-500 (*n=*42) or placebo (*n=*13). In the 150 mg dose panel, only 7 of the 8 possible subjects were treated. The principal investigator decided to stop further dosing after the next to last subject as the 150 mg dose was poorly tolerated (see Table 1). Unblinding revealed that the final subject should have received a placebo. Further, one individual (21-year-old male; 100 mg dose) discontinued prior to study completion because he was lost to follow-up (day 14) after study furlough (day 7). This subject could still be included in the safety and PK analysis as data until day 7 was available. The total dataset comprised 55 subjects for safety and 42 for PK analysis (Fig. 1).

**Table 1:** Summary of adverse events in the single-ascending-dose study (Part A) and the PET study (Part B)

	Single-ascending-dose study									PET study
		CSTI-500 (mg)								CSTI-500 (mg)
Overview	Placebo	0.5	2.5	10	25	50	100	150	Total^a^	100
Subjects treated	13	6	6	6	6	6	6	6	55	9
AEs	8	3	6	5	6	13	7	27	75	15
Subjects with AEs	7	3	3	3	3	4	4	6	33	7
Subjects with SAEs	0	0	0	0	0	0	0	0	0	0
Discontinuations due to AEs	0	0	0	0	0	0	0	0	0	0
Most frequent AEs^b^										
Nausea	0	0	0	0	1	1	1	6	9	4
Dizziness	0	0	1	0	0	2	0	5	8	0
Headache	1	0	0	2	1	2	0	1	7	0
Nasopharyngitis	2	0	2	0	0	0	1	0	5	1
Fatigue	0	1	0	0	1	1	0	2	5	0
Sleep Disorder	1	0	0	0	0	0	0	3	4	2
Dry Mouth	0	0	0	0	1	1	1	1	4	1
Euphoric Mood	0	0	0	1	1	0	1	1	4	0
Decreased Appetite	0	0	0	0	1	0	0	2	3	0

^a^Sum of part A: placebo and dose groups 0.5-150 mg CSTI-500

^b^Categories were based on all AEs reported in at least three subjects in Part A

Abbreviations: *AE* adverse event, *SAE* serious adverse event, *PET* positron emission tomography

#### Safety and tolerability

A summary of the observed AEs is given in Table 1. Notably, there were no serious AEs or study discontinuations due to the CSTI-500 treatment, and all AEs resolved. Of the 55 randomized and dosed subjects, 60.0% experienced AEs during the study: 26 out of 42 subjects treated with CSTI-500 (47.2 %) and 7 out of 13 subjects with placebo (53.8%). All the AEs after a placebo administration were mild. Among the 42 CSTI-500 treated subjects, the intensity of the AEs was judged as mild (*n=*14), moderate (*n=*9), and severe (*n=*3).

For the CSTI-500 treated subjects, the most common AEs were nausea (*n=*9; 21.4%) and dizziness (*n=*8; 19.0%), with the majority in the 150 mg dose panel. Fatigue (*n=*5; 11.9 %), dry mouth (*n=*4; 9.5%), euphoric mood (*n=*4; 9.5%), and decreased appetite (*n=*3; 7.1%) occurred in different dose groups but not in placebo. One subject had a tremor after one single dose of 150 mg CSTI. Headache (*n=*6; 14.3%) and nasopharyngitis (*n=*5; 11.9%) were reported in both placebo and different dose groups. Sleep disorder occurred mainly in the 150 mg dose panel (*n=*3; 7.1%) but once in placebo (*n=*1; 2.4%). After a single dose of 150 mg CSTI-500, 5 out of 6 CSTI-500 treated subjects experienced acute moderate to severe nausea and dizziness, and one subject showed nausea and severe vomiting. Therefore, the 100 mg dose was determined to be the MTD.

There was no consistent evidence of a dose-related increase in heart rate following the administration of CSTI-500 for doses up to 100 mg. However, a marked increase in ECG-derived heart rate from baseline was observed for the 150 mg treatment group (10-15 beats/min between 2-4 h pd). No consistent changes were observed in systolic and diastolic blood pressure between 2-4 h pd for all treatment groups. There were no AEs related to laboratory tests.

#### Pharmacokinetics

The pharmacokinetic parameters of CSTI-500 are summarized for seven single oral doses from 0.5-150 mg in Table 2. The median T_max_ was generally 1-2 h, and the median T_1/2_ was 44-50 h pd. Exposures to CSTI-500 increased in a dose-dependent manner. No significant deviation from dose proportionality was found for C_max_ (0.5-150 mg) and AUC(INF) from 2.5-150 mg. The mean oral plasma clearance (CLT/F) was relatively low (2.3-3.0 L/h). The variability (CV%) was low to moderate (C_max_ 11-33%; AUC(INF) 16-25%).

**Table 2:** Pharmacokinetic parameters for CSTI-500 based on the single-ascending-dose study

	CSTI-500 pharmacokinetic parameters							
Dose panel (mg, *n* = 6)	Geometric mean (CV%) C_max_, (ng/mL)	Median (min, max) T_max_ (h)	Mean (min, max) T_1/2_ (h)	Geometric mean (CV%) AUC_(0-T)_ (ng•h/mL)	Geometric mean (CV%) AUC_(INF)_ (ng•h/mL)	Geometric mean (CV%) C24 (ng•mL)		Geometric mean (CV%) CLT/F (L/h)
0.5	3.60 (19)	2.0 (1, 7)	44 (30, 54)	89.8 (7)	ND		1.73 (7)	3.01 (17)
2.5	24.5 (25)	2.0 (1, 2)	48 (42, 54)	942 (14)	1072 (16)		9.90 (13)	2.33 (17)
10	67.6 (21)	2.0 (2, 2)	50 (34, 72)	2751 (20)	3183 (25)		32.5 (14)	3.14 (28)
25	291 (11)	1.0 (1, 2)	48 (39, 56)	9222 (16)	10455 (16)		105 (23)	2.39 (19)
50	419 (18)	2.0 (1, 5)	45 (40, 54)	14977 (17)	16822 (18)		173 (15)	2.97 (16)
100	870 (33)	1.5 (1, 2)	47 (38, 63)	30649 (20)	34857 (23)		341 (19)	2.87 (18)
150	1334 (24)	2.0 (2, 2)	47 (39, 58)	51980 (19)	58660 (19)		632 (17)	2.56 (20)

Abbreviations: *AUC* Area under the plasma concentration time curve: (1) from zero to the time of the last quantifiable concentration (0-T), and (2) from time zero extrapolated to infinite time (INF); *C24* concentration at 24 h post dosing; *CLT/F* oral plasma clearance; *C*_*max*_ maximum observed plasma concentration; *CV* coefficient of variation, *ND* not determined; *T*_1/2_ apparent plasma elimination half-life; *T*_*max*_ time of maximum observed concentration

### PET study

#### Dataset

The PET study comprised one dose panel investigating the MTD determined in the SAD study, i.e., a single 100 mg dose of CSTI-500. Four subjects were initially enrolled in a SERT and DAT cohort with scheduled PET scans at baseline, 4 and 24 h pd. However, some pd PET scans were rescheduled due to technical difficulties in tracer production (cyclotron breakdown and synthesis failures). Consequently, in the SERT cohort, one subject had a at first pd PET scan at 6 h pd. In the DAT cohort, one enrolled subject withdrew his consent to participate after the baseline scan and was excluded from the dataset (see Fig. 1). Two additional subjects were recruited to achieve at least three observations at 4 and 24 h pd. Finally, the DAT cohort included five subjects with post-dosing [^11^C]PE2I PET scans at 4 h (*n=*3), 9 h (*n=*2), and 24 h (*n=*3) after CSTI-500 administration.

#### Safety and tolerability

Five out of nine subjects experienced AEs related to CSTI-500. Nausea was most common; one subject had a mild AE, two subjects showed a moderate AE, and one subject passed through moderate nausea and severe vomiting within 2 h after dosing. Two other subjects reported sleep disorders, which were judged as moderate. Additional CSTI-500 related AEs were diminished concentration, impaired libido, tremor (all mild), and dry mouth (moderate), each AE occurring only in one subject.

#### Serotonin and dopamine transporter occupancy

**Fig. 2:**
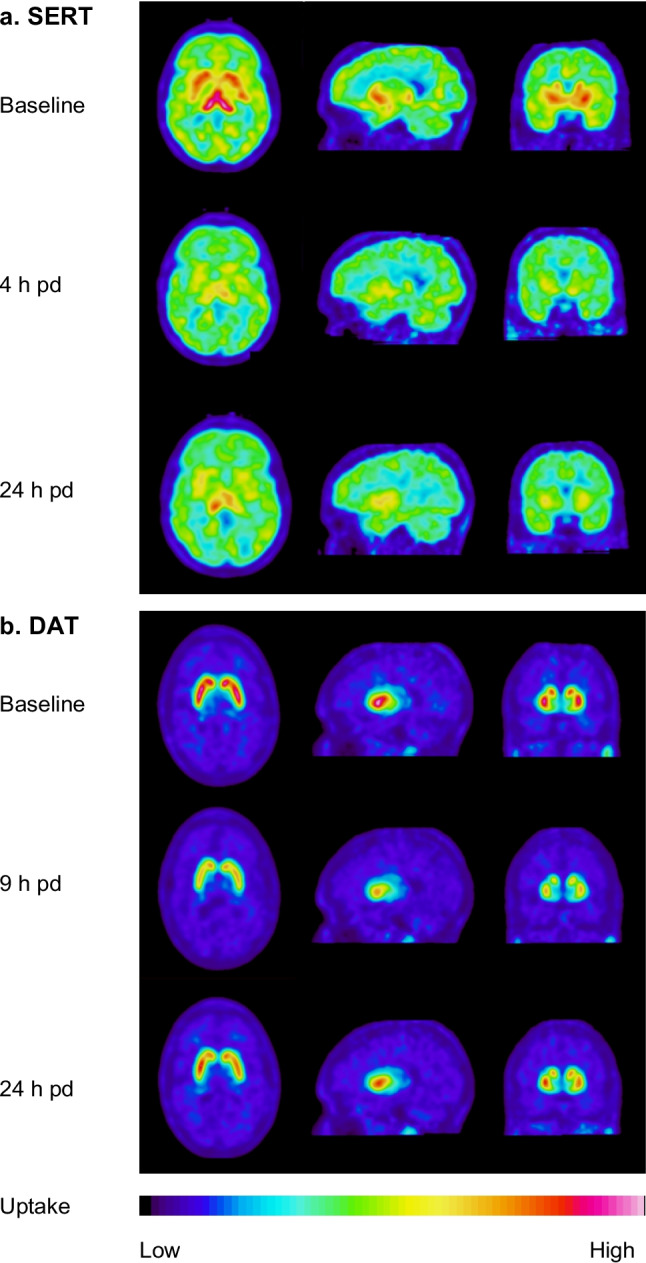
Brain distribution of [^11^C]MADAM (**a**) and [^11^C]PE2I (**b**) targeting serotonin and dopamine transporters, SERT and DAT, respectively, for two different subjects, prior to dosing (baseline) and at two time points after a single-dose of 100 mg CSTI-500. The tracer uptake is illustrated at the level of basal ganglia and expressed relative to the uptake in the cerebellum in a late summation image (30-70 min post-tracer administration)

The brain distribution of [^11^C]MADAM and [^11^C]PE2I is illustrated in Fig. 2 for one representative subject from the SERT and DAT cohort, respectively. The baseline images demonstrate that the [^11^C]MADAM uptake is widespread throughout the brain, although most in central regions (a), while the [^11^C]PE2I uptake is mainly in the striatum (b). Every subject was its own control, and a decrease in tracer uptake after dosing compared to baseline was due to the competitive binding of CSTI-500 to SERT or DAT. The [^11^C]MADAM PET images showed a pronounced reduction of tracer uptake at 4 h pd with a slight decline at 24 h pd. For DAT, CSTI-500 caused a more minor but evident reduction in [^11^C]PE2I uptake at 9 h pd, whereas there was still a notable reduction of the tracer uptake at 24 h pd.

**Fig. 3:**
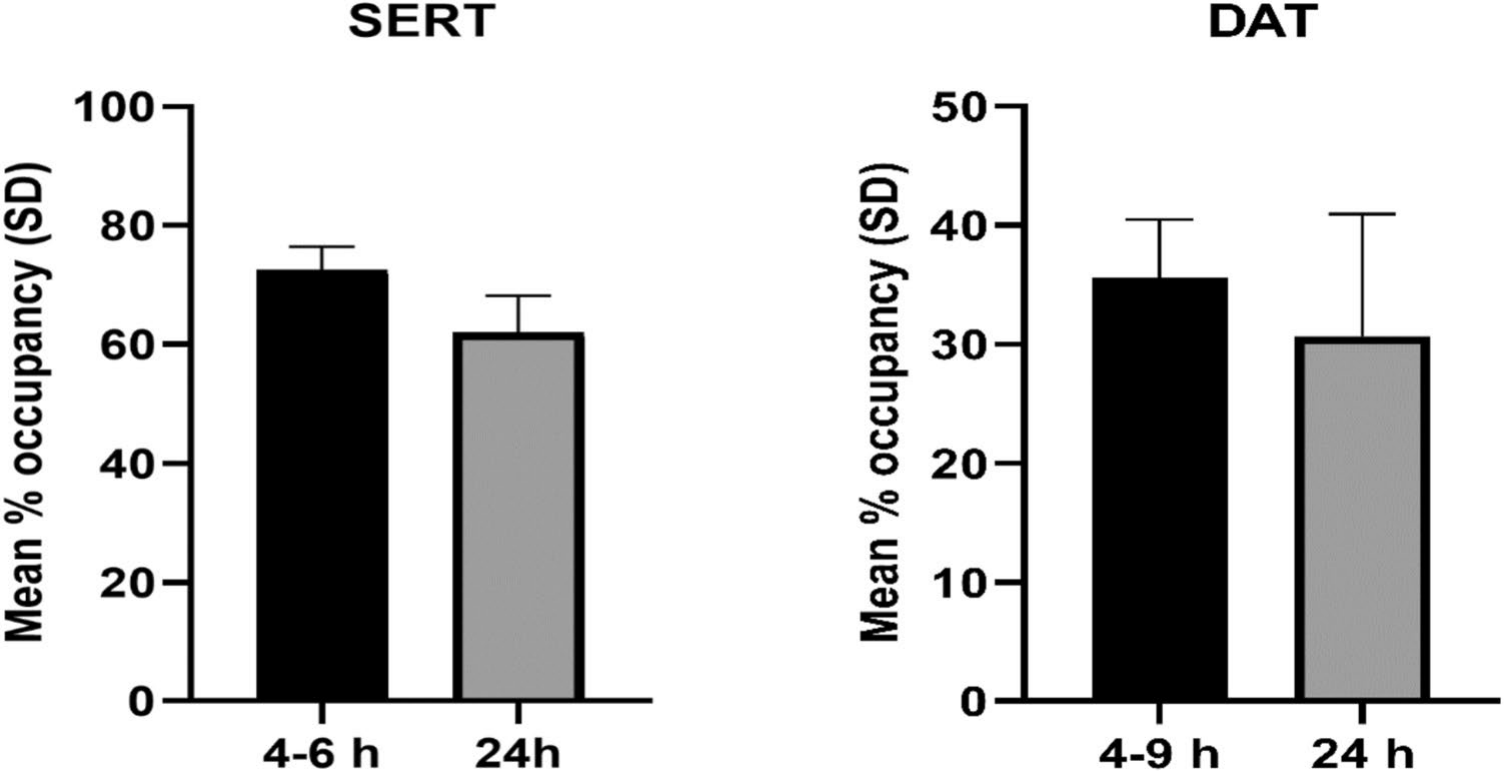
Mean serotonin transporter (SERT; *n=*4) and dopamine transporter (DAT; *n=*5 (4-9 h), *n=*3 (24 h)) occupancies in the striatum following a single oral dose of 100 mg CSTI-500 at 4-9 and 24 h post-dose

Following a single oral dose of 100 mg CSTI-500, the mean striatal SERT occupancies (*n=*4) were 72% (SD 4.2%) and 62% (SD 6.3%) at 4-6 and 24 h pd, respectively (Fig. 3). The mean striatal occupancy values for DAT, were 36% (SD 5.0%; *n=*5) at 4-9 h pd and 31% (SD 10.6%; *n=*3) at 24 h pd. Individual occupancy data are presented in Online Resource 4 for SERT [Supplementary Table 1] and DAT [Supplementary Table 2].

#### Relationship pharmacokinetics and transporter occupancy

**Fig. 4:**
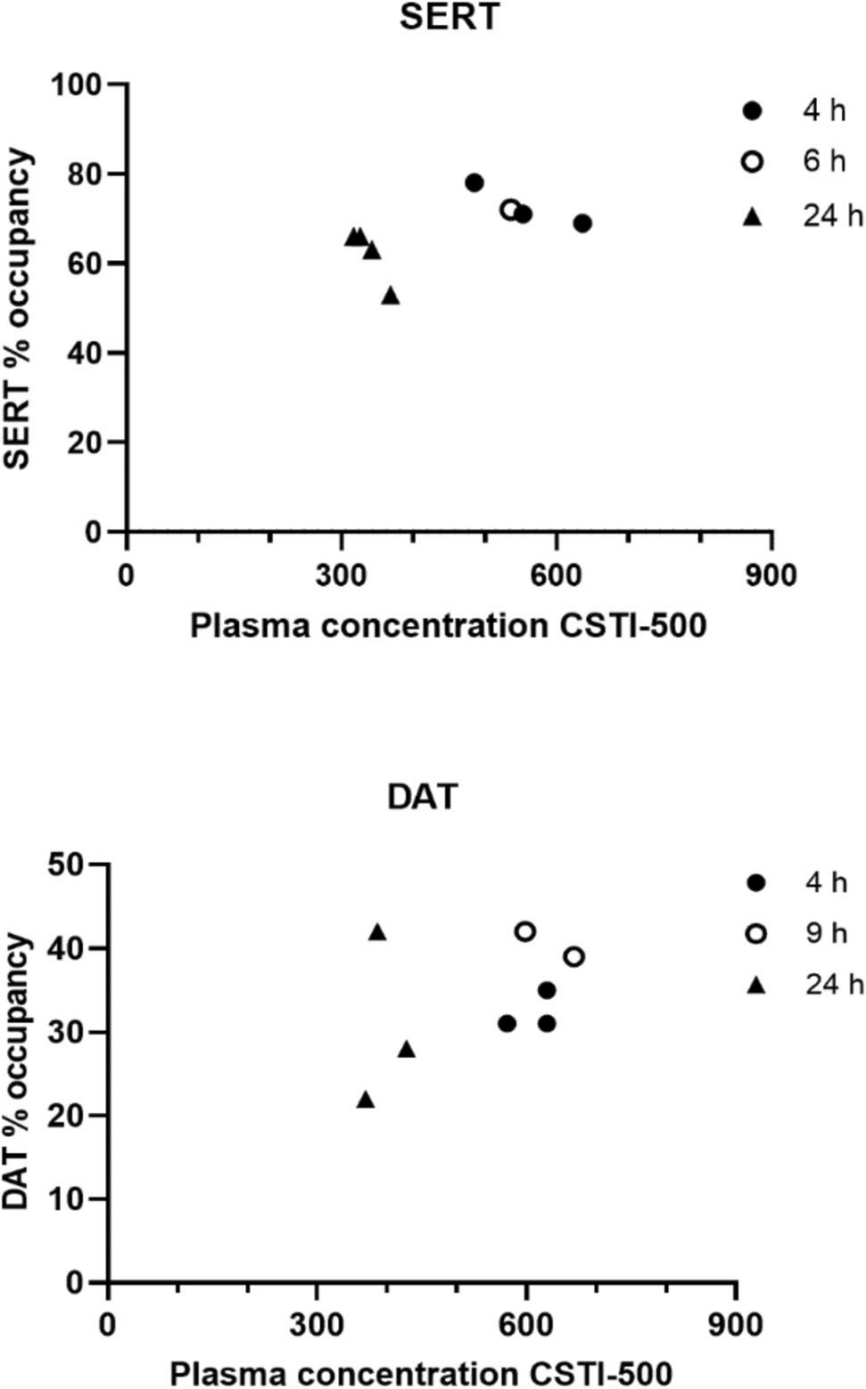
Scatter plots to explore relationships between plasma CSTI-500 concentrations (ng/mL) versus striatal occupancies at serotonin transporters (SERT) and dopamine transporters (DAT) after a single oral dose of 100 mg CSTI-500 at 4-9 and 24 h post-dose

Scatter plots displaying relationships between plasma CSTI-500 concentrations versus striatal SERT/DAT occupancies are presented in Fig. 4. The mean CSTI-500 concentrations for the SERT cohort were 553 and 338 ng/mL at 4-6 and 24 h pd, respectively. In the DAT cohort, the mean CSTI-500 concentrations were 619 and 394 ng/mL at 4-9 and 24 h pd, respectively. The highest striatal DAT occupancies were observed for the two subjects scanned at 9 h pd (39 and 42%). One of these subjects had the same striatal DAT occupancy at 24 h pd, while the CSTI-500 plasma concentration was reduced by about 35%. A non-linear relationship between CSTI-500 plasma concentrations and striatal occupancy levels was indicated for SERT while there was a tendency for a linear relationship for DAT.

## Discussion

### Significance

This work presents the first clinical results of the novel monoamine TRI CSTI-500 based on a single-dose escalation study and a PET study investigating striatal SERT and DAT occupancies for the highest safe and tolerable single-dose, conducted in young, healthy subjects. The SAD study showed that single doses up to 150 mg CSTI-500 were without significant safety concerns in healthy volunteers. However, the 150 mg dose was poorly tolerated, and consequently, the highest safe and tolerable single-dose was determined as 100 mg. The PET study showed that CSTI-500 is a potent monoamine TRI with a pronounced occupancy of SERT and DAT after a single-dose of 100 mg CSTI-500, which was in line with preclinical findings. NET occupancy was not investigated clinically with a PET radioligand, but the occupancy is expected to be approximately aligned with the DAT estimate based on preclinical studies. Pharmacokinetic data revealed that CSTI-500 has a supportive profile for once-daily dosing with rapid oral absorption and low plasma clearance, resulting in a long plasma elimination half-life. The extensive plasma half-life of CSTI-500 and the duration of striatal SERT and DAT occupancies suggest that similar occupancies may be achieved at relatively low doses after repeated daily dosing. Altogether, the results provide a solid foundation for guiding decisions related to the subsequent steps in the clinical development of CSTI-500. Additionally, this clinical study demonstrates the potential of combining safety and tolerability assessments, pharmacokinetics, and monoamine transporter occupancies at an early stage in drug development programs.

### Safety and tolerability

A dose relationship was apparent with the incidence of nausea after administration of 150 mg (*n=*6) and 100 mg CSTI-500 (*n=*4) in the SAD and PET study, respectively. Nausea and vomiting can be triggered by high serotonin and dopamine levels in the CNS and gastrointestinal tract (McManis and Talley 1997; Zhong et al. 2021). The incidence of sleep disorder also indicated a dose relationship for 150 mg (SAD study; *n=*3) and 100 mg (PET study; *n=*2), which in this case might be associated with extensive levels of especially serotonin. However, sleep and wake functions are complex, and disturbances are common side effects of psychotropic as well as non-psychotropic medication (Van Gastel 2018). Although a tremor at rest was only observed in two subjects (150 mg, SAD study; 100 mg, PET study), it is most likely dose-related due to excessive serotonin levels. Approximately 20% of patients treated with selective serotonin reuptake inhibitors (SSRIs) develop tremors without any previous history (Morgan et al 2017). Generally, young, healthy subjects with a functional central neurotransmitter system are more sensitive than untreated patients, and one acute single dose might cause tremors due to a disrupted balance between monoamines. Fatigue and dry mouth are common AEs with the treatment of SERT and NET reuptake inhibitors, whereas euphoric mood and decreased appetite are related to DAT reuptake inhibition.

Adverse cardiovascular effects have been previously associated with drugs having a substantial DAT/NET reuptake inhibition, such as methylphenidate (James et al. 2010; Volkow et al. 2003) and GSK372475 (Learned et al. 2012). This study reported no specific cardiovascular AEs, although increases in heart rate relative to baseline detected at the 150 mg dose level differed notably compared to lower CSTI-500 doses. This lack of cardiovascular AEs suggests a balanced modulation of monoamine inhibitory effects, characterized by moderate DAT occupancy and without excessive norepinephrine activity.

An evident increase in heart rate and blood pressure after food consumption was observed in all treatment groups, including placebo (see Online Resource 1), which was most likely reinforced by a long fasting period (at least 10 h before and 4-5 h after dosing). Notably, this unexpected observation was related to the study design’s operational procedures rather than the study drug. A source for this statement was found in the investigations by Buckhout and Grace (1966). Based on an experimental study in twenty young, male subjects, they concluded that heart rate arousal occurs in food deprived humans anticipating immediate satiation, while those who expect to continue deprivation show less heart rate arousal.

### Pharmacodynamics

The observed striatal SERT and DAT occupancy levels in humans confirmed preclinical findings that CSTI-500 is a serotonin-preferring TRI, and as demonstrated for therapeutic SSRIs (Meyer et al. 2004), a high striatal SERT occupancy is also a desired characteristic of monoamine TRIs. Average striatal SERT peak occupancies were about 70%, nearly twice as high as for DAT. Unexpectedly, the highest striatal DAT occupancies were obtained for the two subjects with their PET scan at 9 h pd. This result may indicate that the peak striatal DAT occupancy in humans was reached later than 4 h pd, although maximum plasma levels were generally observed at 1-2 h pd dose. Some evidence for a later peak of brain DAT occupancy compared to SERT was found *in ex vivo* studies in mice after administration of 1 mg/kg and 5.6 mg/kg of CSTI-500 (data on file). In both cases, the SERT occupancy was nearly constant between 1-8 h pd, but the peak DAT occupancy was achieved at approximately 5 h pd. NET occupancy was not obtained in this clinical study, but *ex vivo* mice experiments demonstrated that CSTI-500 exerted an approximately equipotent reuptake inhibition for DAT and NET. Based on these findings, one single-dose of 100 mg CSTI-500 might result in 30-40% peak NET occupancies. Intermediate DAT and NET occupancies diminish the risks for cardiovascular adverse effects, which were absent for single CSTI-500 doses up to 100 mg.

The estimated striatal DAT occupancies showed some associations with the reported AEs. In the SAD study, the primary dose-limiting AEs were nausea, dizziness, and vomiting, observed after a single-dose of 150 mg CSTI-500. In the PET study, four out of nine subjects reported nausea, and one encountered vomiting after a single-dose of 100 mg CSTI-500. Three individual observations of nausea, including the vomiting case, were made in the DAT cohort, and it was noticed that these subjects had the highest striatal DAT occupancy at 4-9 h pd (35-42%). The other two subjects had a striatal DAT occupancy of 31% at 4 h pd and did not report any AEs. In the SERT cohort, two subjects had drug-related AEs, including nausea and sleep disorders, and two had none. However, there was no direct relationship between striatal SERT occupancy and reported AEs.

For [^11^C]MADAM, the TACs showed slightly higher cerebellar uptake in both post-dosing scans than the baseline scan in all four individuals, probably due to blocking peripheral SERT caused by a single dose of 100 mg CSTI-500. Another [^11^C]MADAM PET occupancy study reported a similar result after single doses of citalopram (Lundberg et al. 2007), and this phenomenon is probably general for all SERT inhibitors. A plausible explanation is an extracerebral, non-displaceable binding of CSTI-500 to SERT, amongst others, to blood platelets (Lundberg et al. 2007) and peripheral organs such as the heart and lungs (Suhara et al. 1996). The modest effect of CSTI-500 on the cerebellar post-dosing TAC data may cause a minor underestimation of SERT occupancies. However, the overall effect is most likely negligible due to the nature of the occupancy equation, thus not significantly affecting the presented results and conclusions for the PET study. The cerebellar [^11^C]PE2I concentrations were not affected by the administration of CSTI-500, and thus, neither did the DAT occupancies.

### Pharmacokinetics

Oral CSTI-500 doses were quickly absorbed for all investigated CSTI-500 doses (1-2 h), but the plasma clearance was relatively low, resulting in a long plasma elimination half-life (about two days). As expected, exposures to various doses of CSTI-500 demonstrated dose proportionality (0.5-150 mg) for plasma concentration measures. The pharmacokinetics partially mirror the pharmacodynamics, i.e., the time course of monoamine transporter occupancies, both striatal SERT and DAT occupancies declined only slightly from 4-9 to 24 h after a single-dose of 100 mg CSTI-500. The extensive plasma half-life and striatal occupancy duration of CSTI-500 point to characteristics for once-daily dosing, and at steady state conditions, similar occupancies might be obtained after relatively low doses. Further, the prolonged effects of CST-500 in plasma and brain are very propitious characteristics as it aids compliance with therapeutic drug concentrations, mitigates against the occurrence of discontinuation symptoms after prolonged therapy, and, most importantly, eliminates the potential for abuse liability (Lane 2015). There was a tendency for a linear relationship between CSTI-500 plasma concentrations and striatal DAT occupancy levels, but no reliable modeling was feasible as we investigated only one dose and had a limited number of data points. Therefore, estimation of EC_50_ and E_max_ will be a valuable issue for following studies with a larger number of data points, and preferably, several doses of CSTI-500.

### Insights from PET occupancy studies

Most monoamine TRIs have initially been developed as an antidepressant for MDD. However, several clinical development programs for potential monoamine TRI antidepressants have failed to demonstrate significantly greater efficacy than placebo or standard of care. For the successful development of a monoamine TRI, two critical factors must be considered: (1) the optimal balance of relative reuptake inhibitory potencies for the three monoamine transporters, and (2) the selection of an appropriate target patient population that would benefit from these effects (Lane 2015). Due to synergic or additive effects, the optimal balance of relative reuptake inhibitory potencies for the three monoamine transporters might be different from the clinically effective reuptake inhibition at each transporter in single or dual reuptake inhibitors (Learned et al. 2012). From a safety perspective, excessively high inhibitory potencies for the NET and DAT are undesirable due to potential adverse cardiovascular effects. Knowledge of the balance of relative monoamine transporter potencies in humans is preferably obtained early in the drug development process using PET with highly selective tracers. A keystone in the development of antidepressants is a high striatal SERT occupancy. Meyer and colleagues (2004) showed that for five efficacious SSRIs targeting treatment for depression, the striatal SERT occupancy was approximately 80% and accomplished after a 4-week administration of minimum therapeutic daily doses but clinically distinguishable from placebo. Therefore, novel monoamine TRI drug candidates developed for MDD may be selected first based on a high striatal SERT occupancy. After that, the optimal combination of NET and DAT reuptake inhibition must be established. Several clinical studies have shown that balancing inhibitory potencies is challenging despite promising preclinical results.

Two subsequent 10-week phase II trials in MDD patients demonstrated that the monoamine TRI GSK372475 (1-2 mg/d) was neither efficacious nor well-tolerated (Learned et al. 2012). The reported AEs and cardiovascular data suggested that tolerability was affected due to high DAT and NET occupancies. The doses were based on various phase I studies, including a PET occupancy study in healthy subjects, and judged as safe and tolerable. SERT and DAT occupancies ranged from 55-75% and 60-80%, respectively, while preclinical data indicated a similar potency for NET. Unbalanced ratios of monoamine potencies can explain the lack of tolerability and, in part, efficacy. A lower dose (0.5 mg/d) to increase tolerability in MDD patients was no alternative, as it presumably resulted in SERT occupancies far below 80%.

The efficacy and safety of the monoamine TRI BMS-820836 (0.25-2 mg/d) were investigated in patients with treatment-resistant MDD in two phase IIb trials (Bhagwagar et al. 2015). Although the doses were well-tolerated in patients, they showed no clinical superiority compared to duloxetine or escitalopram treatment. The design was based on the results from a previous multiple-ascending-dose study (MAD) in phase I, including safety, tolerability, pharmacokinetics, and pharmacodynamics using PET, in healthy subjects. BMS-820836 was generally safe and well tolerable, reaching about 80% SERT occupancy by a relatively low dose (0.5 mg/d) near steady-state conditions (Zheng et al. 2015). The lack of efficacy could partially depend on the relatively low striatal DAT occupancies of 14-30% (0.5-2 mg/d) in healthy volunteers. Higher doses were not feasible as there was a linear dose-related escalation in heart rate. Blood pressure also increased but without a consistent dose-response pattern. Consequently, there was an enhanced risk for cardiovascular adverse effects, most likely based on NET inhibition, which could result in disproportionate amounts of norepinephrine.

A PET occupancy study with different single-doses of the monoamine TRI SEP-225289 (8-16 mg) in healthy volunteers (DeLorenzo et al. 2011) demonstrated a low SERT occupancy (mostly below 20%) for all doses, thus predicting a weak antidepressant effect. This result confirmed the outcome of an earlier phase II study in MDD patients where no reduction of depression symptoms was found after 8 weeks of treatment (Sharma et al. 2015). In contrast, DAT occupancies demonstrated a dose-dependency with average values between 33-49%. NET was not evaluated. GSK136077, also a monoamine TRI developed for MDD, demonstrated SERT occupancies from about 20-80% and DAT occupancies from 20-60% in healthy volunteers after one single administration of various doses (Comley et al. 2013). Investigations in baboons indicated GSK136077 to be most potent in NET. However, the high potency for all targets and linear dose dependencies in occupancy might imply that multiple doses of GSK136077 will be associated with unacceptable safety and tolerability. Our PET study shows that CSTI-500 is a serotonin-preferring TRI with relatively high SERT and moderate DAT occupancies after a single-dose of 100 mg. It is predicted that a high SERT occupancy can be reached at a relatively low dose in a multiple-dose setting. This result may allow “dialing in” DAT and NET occupancy by gradual dose escalation where the exposure increases but not to levels that jeopardize safety.

In summary, these monoamine TRI PET studies show a wide variation in reported central SERT and DAT occupancies. Although less objectively quantified, variation in NET occupancy is also evident. All compounds were initially aimed for development as novel antidepressants based on promising preclinical experiments but have been discontinued or are targeting other patient groups. However, the historical PET occupancy data reveals that the balance of monoamine occupancies needs further investigation. Drug development programs could be further optimized and lead to decreased risks by assuring that monoamine transporter’s approximate target occupancy were met before phase II and later clinical trials are conducted (Comley et al. 2013; Lane 2015; Sharma et al. 2015). Further, while the central SERT and DAT occupancies are well investigated for established pharmaceuticals in CNS disorders, less is known about the central NET occupancy. The absence of knowledge on NET occupancy hampers discovering the optimal combination of reuptake inhibition via transporter blockades for monoamine TRIs in targeted patient groups. However, in recent years, some clinical studies report NET occupancies (Arakawa et al. 2019; Matuskey et al. 2023) due to the development of novel CNS NET targeting radiotracers and advances in PET scanner technology (Chen et al. 2020) as well as evolved quantification methods. These new insights achieved by PET may provoke further investigation of the effectiveness of monoamine TRIs in MDD and other neuropsychiatric disorders.

### Future perspectives in clinical drug development

Clinical development programs for MDD are associated with significant risks as they require extensive logistical and financial resources. Less common or rare disorders with dysregulated monoamine systems marked by significant unmet medical needs present feasible target populations for drug development in small pharmaceutical companies as they demand less resources and consequently imply lower risks. The hallmark symptom in individuals with Prader-Willi Syndrome (PWS) and hypothalamic injury-induced obesity is hyperphagia or insatiable appetite due to hypothalamic dysfunction, which can lead to extreme obesity. The monoamine TRI tesofensine was not efficacious for treating Alzheimer’s and Parkinson’s diseases but induced a significant weight loss as an AE during clinical trials for Parkinson’s disease (Astrup et al. 2008). Interestingly, the results of a phase I PET study in healthy subjects suggested that an optimal therapeutic effect of tesofensine in obese subjects could be reached with doses of 0.5-1 mg, resulting in a striatal DAT occupancy between 50 and 70% at steady-state conditions (Appel et al. 2014). At a later stage, tesofensine was reformulated to Tesomet, a fix-dose combination with metoprolol, to address cardiovascular effects. In a phase IIa exploratory study, Tesomet showed strong efficacy on hyperphagia and weight with once-daily Tesomet (0.5 mg tesofensine; 50 mg metoprolol) (Torok et al. 2019). Recently, Huynh et al. (2022) reported a significant reduction in body weight in patients with hypothalamic-injury-induced obesity compared to placebo after a 24-week treatment with Tesomet once daily (0.5 mg tesofensine; 50 mg metoprolol). In addition to hyperphagia, almost all individuals with PWS suffer from behavioral problems such as temper outbursts. Recent research showed that treatment of PWS patients with SSRI sertraline for 6 months significantly reduced the temper outbursts in an open-label study at doses as low as 25 mg (Deest et al. 2021), which would correspond to approximately 70% of striatal SERT occupancy (Meyer et al. 2004). Based on the previous findings above, it might be that CSTI-500 has unique properties by having the potential for treating hyperphagia, obesity, and temper outbursts in patients with PWS and hypothalamic injury-induced obesity. A phase II study of CSTI-500 in PWS is, however, demanded to confirm the pharmaceutical potential of CSTI-500 for this patient group.

### Study limitations

This clinical trial, though relatively small, is significant due to combining a SAD phase I trial with a PET study. This unique approach delivers beneficial supplementary information for a first-in-human study. However, caution should be exercised in generalizing results in young, healthy volunteers directly to the intended target patient groups. For example, young, healthy subjects have fully functional central monoaminergic systems and are more sensitive to the adverse effects of monoamine re-uptake inhibitors than unmedicated MDD patients with a blunted monoamine activity (Newberg et al. 2012; Pizzagalli et al. 2019). Moreover, in clinical practice, there is invariably gradual titration to higher doses to mitigate the acute adverse effects of these drugs. Further, only women aged 18-55 years without child-bearing potential were allowed to participate in the SAD study due to the lack of preclinical reproductive toxicology data. This led to a disruption in the balance between sexes in the SAD study, with low female participation (Fig. 1), which can potentially affect the interpretation of safety and tolerability results. Additionally, the cardiovascular safety data from about 5 h pd were noticeably affected in all treatment groups, most likely due to food consumption and a long fasting period. These operational procedures should be a point of attention for the design of phase I studies. However, we are confident in using an interval of 2-4 h pd for evaluating acute effects on vital signs after a single-dose of CSTI-500. In the PET study, only men were included as possible sex effects could increase the variation in occupancy results with relatively small cohorts and consequently impede its interpretation. However, the results of the PET study might vary between sexes, which should be investigated in future studies, particularly the dose-occupancy relationship. Notably, only one dose, the MTD of CSTI-500, was investigated in the PET study. Another panel examining striatal occupancies for lower CSTI-500 doses might have provided additional information for exploring the PK-SERT/DAT occupancy relationship and enhanced guidance for daily dosing of CSTI-500 in a multiple-dose scenario. Although these limitations may restrict the interpretation of the results and the translation to the entire population of a defined patient group, the outcome measures highly support the continued clinical development of CSTI-500.

## Conclusions

This first-in-human SAD study showed that CSTI-500 is a potent monoamine TRI with promising safety, tolerability, and pharmacokinetics profiles (0.5-150 mg), as well as favorable pharmacodynamics, including time course of monoamine transporter occupancies. There were no serious safety concerns, although heart rate showed a marked increase after a single-dose of 150 mg CSTI-500. The maximum tolerable single-dose of CSTI-500 was determined as 100 mg. Oral CSTI-500 doses were quickly absorbed and had a relatively low plasma clearance, resulting in a long plasma elimination half-life (about two days). CSTI-500 showed characteristics of a serotonin-preferring monoamine TRI after a single-dose of 100 mg with peak striatal occupancies of about 70%, nearly twice as high for DAT, followed by a slight decline at about 24 h pd. The absence of serious adverse cardiovascular effects suggests a balanced modulation of monoamine inhibitory effects, characterized by moderate DAT occupancy and without excessive norepinephrine activity. Based on the slow pharmacokinetics and pharmacodynamics of CSTI-500, similar occupancies may be obtained with relatively low CSTI-500 doses when using a repeated once-daily dosing treatment. Taken together, these encouraging results strongly support the continued clinical development of CSTI-500, but it is important to evaluate further this monoamine TRI with multiple dosing regimens and use of PET imaging to characterize its properties at steady-state conditions in human.

## Supplementary Information

Below is the link to the electronic supplementary material.

Supplementary file1 (DOCX 1336 KB)

## Data Availability

Investigator inquiries on data availability should be submitted to Consynance Therapeutics Inc., see https://www.consynance.com. Requests will be judged on reasonability and applicable regulatory requirements.
