# Supplementary material for: Ascending single-dose study of the safety, pharmacokinetics, and pharmacodynamics of CSTI-500, a novel monoamine triple reuptake inhibitor, first-in-human
Source: Psychopharmacology (Berl). 2025 Aug 11;243(3):565–80. doi: 10.1007/s00213-025-06861-4 (PMC12979414; doi:10.1007/s00213-025-06861-4)
Supplement: Supplementary file 1 — Supplementary file1 (DOCX 1336 KB) [file 213_2025_6861_MOESM1_ESM.docx]

**ONLINE RESOURCES** (Electronic supplementary material)

**Article title:** Ascending single-dose study of the safety, pharmacokinetics, and pharmacodynamics of CSTI-500, a novel monoamine triple reuptake inhibitor, first-in-human

**Journal name:** *Psychopharmacology*

**Author names:** Lieuwe Appel, Robert Risinger, Anders Wall, Harald Murck, Shuang Liu, Gunnar Antoni, Roger Lane

**Corresponding author:** Lieuwe Appel**;** PET Centre, Department of Medical Imaging, Uppsala University Hospital, 751 85 Uppsala, Sweden; [Lieuwe.Appel@akademiska.se](mailto:Lieuwe.Appel@akademiska.se)

**Online Resource 1 – Cardiovascular Safety Evaluation (incl. [Fig S1-S3])**

**Background**

Heart rate (HR) and blood pressure (BP) are part of the core battery assessments for cardiovascular safety evaluation of a new chemical entity, following ICH guidelines for Good Clinical Practice. In the single-ascending-dose (SAD) study, the subjects’ HR and BP profiles were recorded continually using an ambulatory monitor (Spacelabs 90207 ABPM device), with data collection every 30 min post-dosing (pd), compared to a baseline measurement (time of dosing, 0 h). Monitoring took place to 15 h pd. The ABP monitor contains an algorithm that performs data error checks on the HR and BP measurements. In the case of an error, the data point at the time of the error was not used. If an error occurred, the ABP monitor automatically attempted another reading approximately two minutes later, which was the case for approximately 8% of readings. The ABPM devices were removed prior to the subjects’ bedtime to avoid interference with the subjects’ sleep.

In the SAD study, seven panels of eight healthy subjects were enrolled and administered a single-dose of CSTI-500 ranging from 0.5 to 150 mg. In each panel, six subjects received the study drug, and two received a placebo. However, only one subject received placebo in the 150 mg panel. Subjects were fasting for at least ten hours before dosing. The 0.5 mg panel was completed before the installation of ABPM, and consequently, no data from this panel is presented.

Besides HR, systolic and diastolic BP were the cardiovascular safety outcomes. However, after the data collection and completion of the study, we observed a peak of the mean change from baseline on two occasions, particularly for heart rate, but also notable for the BP measurements. This pattern was consistent across all CSTI-500 treatments and placebo administration, leading us to question the operational procedures. The first peak at about 5.5 h pd could be related to the lunch and the one at 10 h pd to the evening dinner. Additionally, a long fasting period of approximately 15 h might have amplified the arousal in HR and BP at 5.5 h pd. Our explanation is supported by [Buckhout and Grace's investigations (1966)](#_ENREF_9). Based on an experimental study in twenty young, male subjects, they concluded that HR arousal occurs in food-deprived humans anticipating immediate satiation, while those who expect to continue deprivation show less HR arousal.

Figures S1-S3 show the linear plots of the mean change from baseline for HR, systolic BP (SBP), and diastolic BP (DBP) with three different time intervals. To ensure the validity of our evaluation, we decided to use only data from stable fasting conditions (2-4 h pd) to investigate the acute effect of CSTI-500 on CV effects.

**Reference**

Buckhout, Robert, and Terrence Grace. 1966. 'The effect of food deprivation and expectancy on heart rate', *Psychonomic Science*, 6: 153-54.

**Figure S1.** Linear plot of mean change from baseline for heart rate (HR) post-treatment in beats per minute (bpm): **(A)** stable fasting conditions after treatment, but before food supply, 2 to 4 h; **(B)** early stage, 0.5 to 7 h with a peak at about 5.5 h after lunch; **(C)** 0.5 to 12 h with peaks at about 5.5 and 10.5 h after lunch and dinner

**Figure S2.** Linear plot of mean change from baseline for systolic blood pressure (SBP) post-treatment: **(A)** stable fasting conditions after treatment, but before food supply, 2 to 4 h; **(B)** early stage, 0.5 to 7 h with a peak at about 5.5 h after lunch; **(C)** 0.5 to 12 h with peaks at about 5.5 and 10.5 h after lunch and dinner

**Figure S3.** Linear plot of mean change from baseline for diastolic blood pressure (DBP) post-treatment: **(A)** stable fasting conditions after treatment, but before food supply, 2 to 4 h; **(B)** early stage, 0.5 to 7 h with a peak at about 5.5 h after lunch; **(C)** 0.5 to 12 h with peaks at about 5.5 and 10.5 h after lunch and dinner

**Online Resource 2 – Time activity curves (incl. [Fig S4-S5])**

Figure S4 Average time activity curves, at baseline conditions (n=4), showing the standardized uptake values (SUV) of [^11^C]MADAM over time for the target region striatum (str) as well as for the reference region cerebellum (cbl)

Figure S5 Average time activity curves, at baseline conditions (n=5), showing the standardized uptake values (SUV) of [^11^C]PE2I over time for the target region striatum (str) as well as for the reference region cerebellum (cbl)

**Online Resource 3 – Time activity curves (incl. [Fig S6-S7])**

| **Fig S6** Kinetic modelling for subject 00111 illustrating the fit of the cerebellar and striatal [^11^C]MADAM time activity data using a simplified reference tissue model at baseline, and 4 and 24 h after administration of CSTI-500 | | |
| --- | --- | --- |
| **Baseline** | **Cerebellum** | **Striatum** |
| 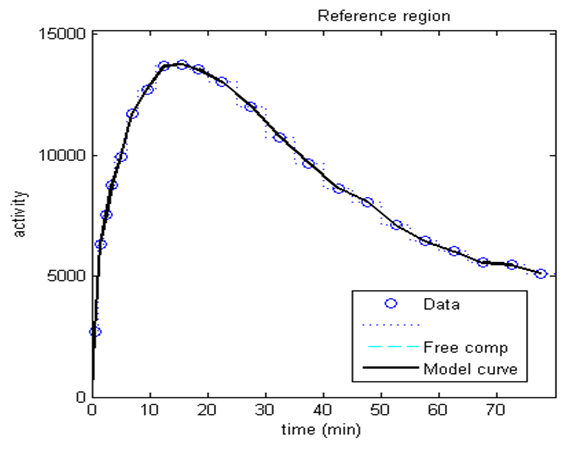 | | 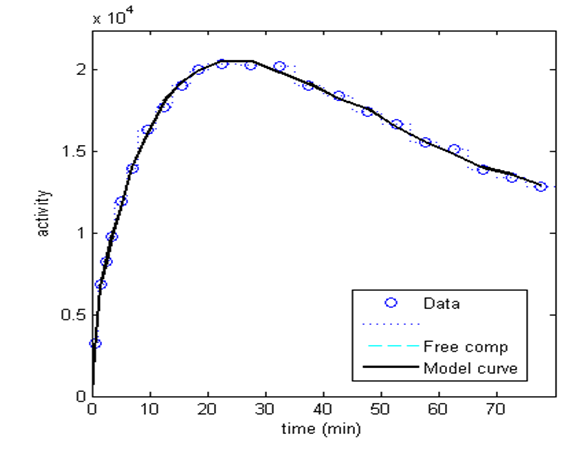 |
| **4 h pd** |  |  |
| 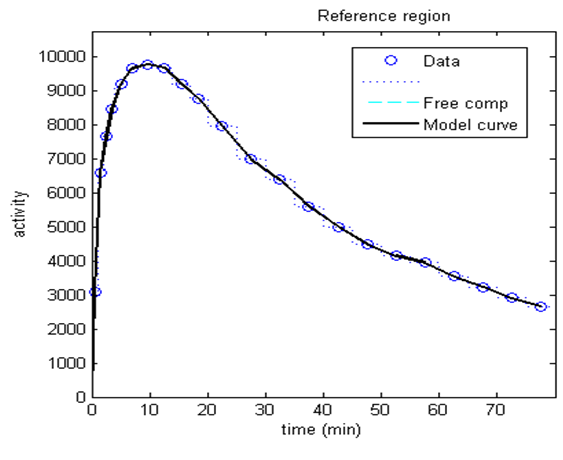 | | 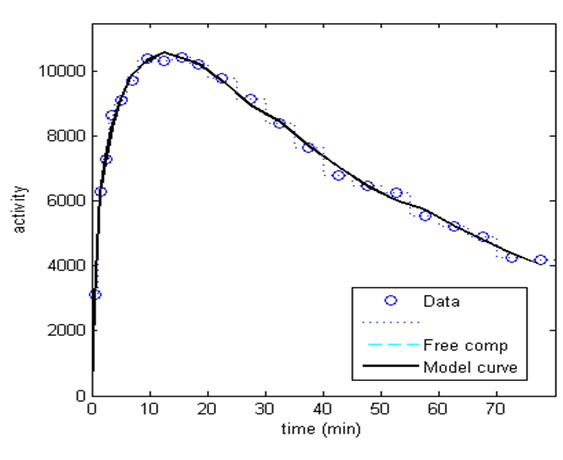 |
| **24 h pd** |  |  |
|  |  |  |
| 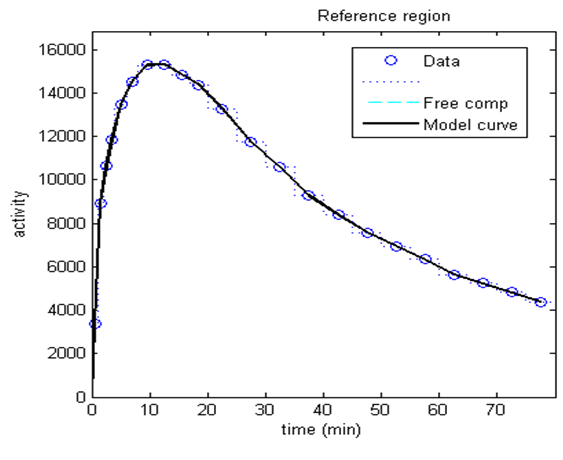 | | 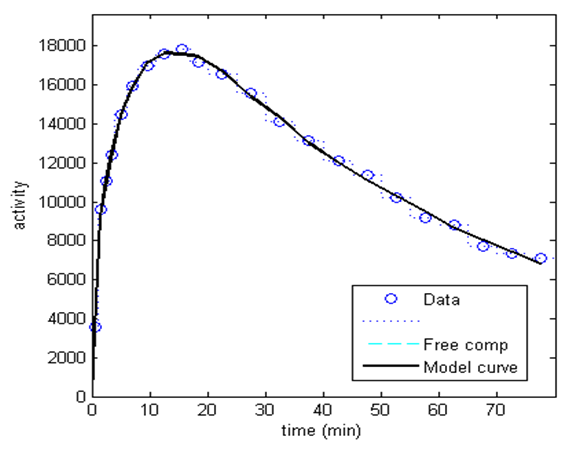 |

**Fig S6** Kinetic modelling for subject 00105 illustrating the fit of the cerebellar and striatal [^11^C]PE2I time activity data using a simplified reference tissue model at baseline, and 9 and 24 h after administration of CSTI-500

|  | | | |
| --- | --- | --- | --- |
| **Baseline** | **Cerebellum** | | **Striatum** |
| 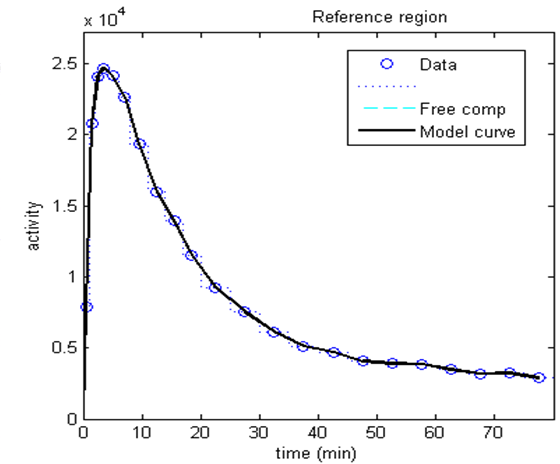 | | 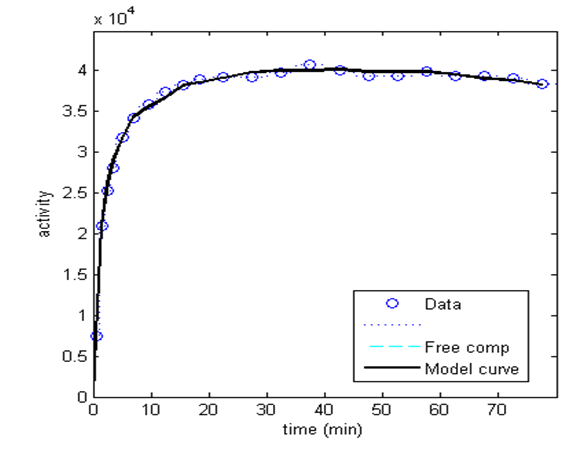 | |
| **9 h pd** |  |  | |
| 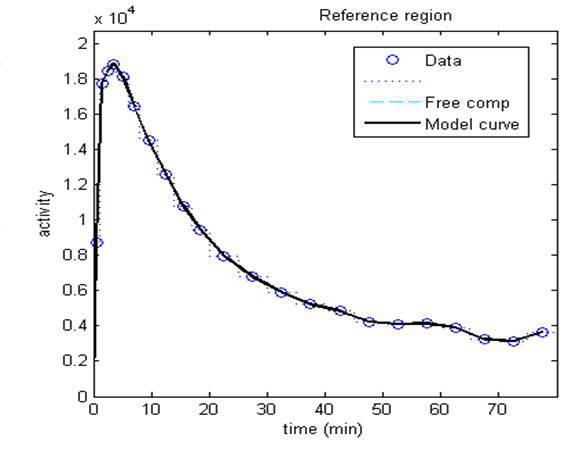 | | 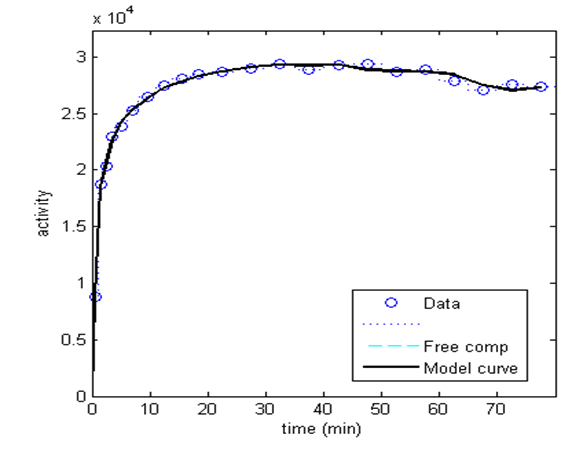 | |
| **24 h pd** |  |  | |
| 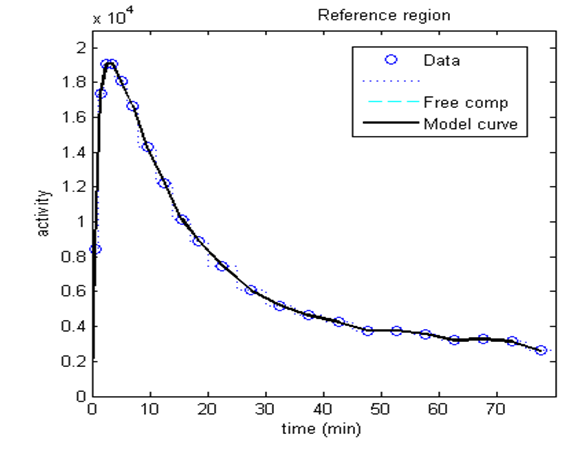 | | 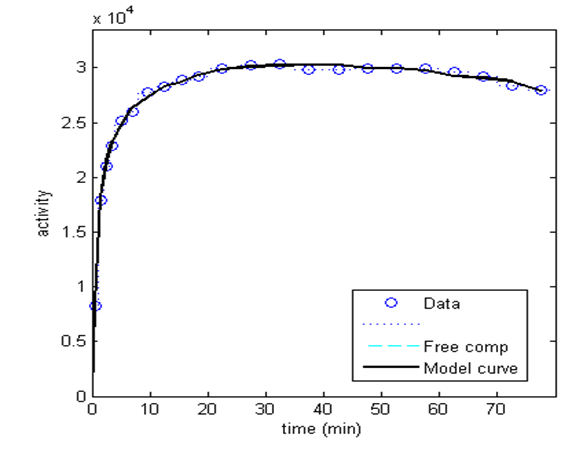 | |

**Online Resource 4 – Individual occupancy estimates (incl- [TablesS1-S2]**)**.**

**Table S1** Striatal serotonin transporter – non-displaceable binding potential (BP_ND_) and occupancy (Occ) following a single-dose of 100 mg CSTI-500

|  |  | **Baseline** |  | **4 hours post-dose** | |  | **24 hours post-dose** | |
| --- | --- | --- | --- | --- | --- | --- | --- | --- |
| **Subject** |  | **BP_ND_** |  | **BP_ND_** | **Occ** |  | **BP_ND_** | **Occ** |
| 00102 |  | 1.22 |  | 0.35 | 72^a^ |  | 0.57 | 53 |
| 00110 |  | 1.30 |  | 0.40 | 69 |  | 0.48 | 63 |
| 00111 |  | 1.14 |  | 0.33 | 71 |  | 0.38 | 66 |
| 00113 |  | 1.09 |  | 0.24 | 78 |  | 0.37 | 66 |
| *Mean* |  | *1.19* |  | *0.33* | *72* |  | *0.45* | *62* |
| *SD* |  | *0.09* |  | *0.07* | *4.2* |  | *0.10* | *6.3* |

^a^ Scan performed at approximately 6 hours post-dose.

**Table S2.** Striatal dopamine transporter – non-displaceable binding potential (BP_ND_) and occupancy (Occ) following a single-dose of 100 mg CSTI-500

|  |  | **Baseline** |  | **4 hours post-dose** | |  | **24 hours post-dose** | |
| --- | --- | --- | --- | --- | --- | --- | --- | --- |
| **Subject** |  | **BP_ND_** |  | **BP_ND_** | **Occ** |  | **BP_ND_** | **Occ** |
| 00101 |  | 9.56 |  | 6.62 | 31 |  | ND^a^ | ND^a^ |
| 00104 |  | 9.08 |  | 6.24 | 31 |  | 7.10 | 22 |
| 00105 |  | 9.69 |  | 5.88 | 39^b^ |  | 7.01 | 28 |
| 00109 |  | 10.77 |  | 6.98 | 35 |  | ND^a^ | ND^a^ |
| 00112 |  | 10.52 |  | 6.08 | 42^b^ |  | 6.07 | 42 |
| *Mean* |  | *9.92* |  | *6.36* | *36* |  | *6.73* | *31* |
| *SD* |  | *0.70* |  | *0.44* | *5.0* |  | *0.57* | *10.6* |

^a^ ND, not determined – scans were not performed due to technological difficulties.

^b^ Scan performed at approximately 9 hours post-dose.
